# Hydrodynamic Voltammetry as a Rapid and Simple Method for Evaluating Soil Enzyme Activities

**DOI:** 10.3390/s150305331

**Published:** 2015-03-04

**Authors:** Kazuto Sazawa, Hideki Kuramitz

**Affiliations:** 1 Center for Far Eastern Studies, University of Toyama, Gofuku 3190, 930-8555 Toyama, Japan; 2 Department of Environmental Biology and Chemistry, Graduate School of Science and Engineering for Research, University of Toyama, Gofuku 3190, 930-8555 Toyama, Japan; E-Mail: kuramitz@sci.u-toyama.ac.jp

**Keywords:** soil enzymes, hydrodynamic voltammetry, rotating disk electrode, microdroplet sample, humic acid, *p*-aminophenol

## Abstract

Soil enzymes play essential roles in catalyzing reactions necessary for nutrient cycling in the biosphere. They are also sensitive indicators of ecosystem stress, therefore their evaluation is very important in assessing soil health and quality. The standard soil enzyme assay method based on spectroscopic detection is a complicated operation that requires the removal of soil particles. The purpose of this study was to develop a new soil enzyme assay based on hydrodynamic electrochemical detection using a rotating disk electrode in a microliter droplet. The activities of enzymes were determined by measuring the electrochemical oxidation of *p*-aminophenol (PAP), following the enzymatic conversion of substrate-conjugated PAP. The calibration curves of β-galactosidase (β-gal), β-glucosidase (β-glu) and acid phosphatase (AcP) showed good linear correlation after being spiked in soils using chronoamperometry. We also performed electrochemical detection using real soils. Hydrodynamic chronoamperometry can be used to assess the AcP in soils, with a detection time of only 90 s. Linear sweep voltammetry was used to measure the amount of PAP released from β-gal and β-glu by enzymatic reaction after 60 min. For the assessment of soil enzymes, the results of hydrodynamic voltammetry assay compared favorably to those using a standard assay procedure, but this new procedure is more user-friendly, rapid and simple.

## 1. Introduction

Enzymes are released in soils by microbes and plant roots. They can degrade complex substrates into low molecular weight compounds in soils [1,2]. The enzymes can be affected by many factors, biological (microbial populations, fauna, *etc.*), chemical (pH, organic matter contents, *etc.*) and, human activities (fire treatment, pesticides, heavy metals, *etc.*) [3,4,5], all of which change the biogeochemical cycles in an ecosystem. Therefore, the evaluation of enzyme activity in the soil is very important in order to assess the biochemical function, organic matter fraction, nutrient cycle, and decomposition of xenobiotics.

In general, regulatory soil enzyme assays are based on the colorimetric determination of *p*-nitro-phenol (PNP) that is released by enzyme reactions when a soil sample is incubated in an optimum buffer solution containing substrate-conjugated PNP at optimum temperature [6,7,8]. However, regulatory assays are not suitable to evaluate a sample that contains a high concentration of colored dissolved organic matter, because these assays are based on spectroscopic detection. Furthermore, regulatory assays require long enzymatic reaction times (1 to 24 h) with substrates and involve complicated methods for soil particle removal by filtration or centrifugation.

The aim of the present study was to develop a new rapid and simple soil enzyme assay based on hydrodynamic electrochemical detection using a rotating disk electrode (RDE) in a microliter (ca. 50 μL) droplet. Soil enzyme activities are determined by measuring the electrochemical oxidation of PAP, which is converted from the substrate by the enzyme reaction in soil. Compared with spectroscopic detection, the advantage of electrochemical detection is that it is much simpler and does not interfere with the colored components and soil particles in a sample solution. Furthermore, hydrodynamic voltammetry in a droplet can achieve the effective mixing of a sample solution and convective mass transport of an analyte to the electrode surface, which results in rapid detection [9,10,11]. Previously, we developed a new genotoxicity test based on hydrodynamic electrochemical detection using a RDE in a microliter droplet, which evaluates the DNA damage caused by chemicals based on the β-gal activity of a bacteria strain [12]. This methods can be used for a rapid assessment of the β-gal activity of a bacteria strain in sediments.

The two main objectives for this study were: (1) to use commercial enzyme activities with or without the soil samples to investigate the use of hydrodynamic chronoamperometry to evaluate the effect of dissolved organic matter and soil particles on the amperometric response of PAP; and (2) to demonstrate the use of the new method in assaying for β-gal, β-glu and AcP activities in real soils and compare the results with the standard assay based on spectroscopic detection. These enzymes are widely distributed in the environment and have been evaluated by several soil scientists. β-gal and β-glu play significant roles in the breakdown of low molecular-weight carbohydrates and cellulose in the soil, and are excreted mainly by microorganisms, animals and plants [13,14,15]. AcP is a major product of plant roots, and breaks down organic P for release in soils [16,17,18,19].

## 2. Experimental Section

### 2.1. Chemicals

Modified universal buffer (MUB) with the desired pH for each of the enzymes was prepared according to previous reports [20,21]. Calcium chloride, PNP, toluene, NaOH, kaolin and quartz sand were purchased from Wako Pure Chemical Industry Ltd. (Osaka, Japan). *p*-Nitrophenyl-β-D-galactopyranoside (PNPGal), *p*-nitrophenyl-β-D-glucopyranoside (PNPGlu), *p*-nitrophenyl phosphate disodium salt hexahydrate (PNPP), *p*-aminophenyl-β-D-galactopyranoside (PAPGal), *p*-aminophenyl-β-D-glucopyranoside (PAPGlu), *Aspergillus oryzae* β-galactosidase (β-Gal; EC 3.2.1.23), almond β-glucosidase (β-Glu; EC 3.2.1.21), potato acid phosphatase (AcP; EC 3.1.3.2), PAP, and humic acid were obtained from Sigma-Aldrich (St. Louis, MO, USA). Humic acid represents a main fraction of soil organic matter. An Aldrich humic acid (AHA) was dissolved in 0.1 M NaOH, and the solution was then treated with a mixture of HF and HCl. The resultant precipitate was transferred to a dialysis tube (molecular weight cutoff 500 Da). After dialysis, the slurry was freeze dried. The purified AHA stock solution (500 mg·L^−1^) was dissolved in 1 M NaOH solution (pH > 10) by stirring for 30 min under a gentle N_2_-stream. The acidity of the solutions was adjusted to pH 8.0 using 1 M HCl. *p*-Aminophenyl phosphate monosodium salt hydrate (PAPP) was obtained from Apollo scientific Ltd. (Stockport, UK). Tris (hydroxymethyl) aminomethane (TMAH) was purchased from MP Biomedicals (Aurora, OH, USA). *Sphagnum* moss peat was purchased from a local market in Toyama, Japan. Kaolin and quartz sand were ground into a fine powder using a mortar and pestle, and passed through a 0.025 mm mesh sieve.

### 2.2. Soil Samples

Two soils were used in the present study. Soil A was collected from agricultural land at the University of Toyama. Soil B was collected from a forest in Toyama, Japan. The soils were sampled from a depth of 0–10 cm, and were air-dried at room temperature, ground for passage through a 2 mm mesh, and stored at 4 °C prior to use. The soils characteristics are presented in Table 1.

**Table 1:** Chemical and physical characteristics of soils used in this study.

	Land Use	pH	EC * (μS/cm)	Moisture Content (%)	Ignition Loss (%)	Abs. at 400 nm ^**^ (TMAH)	Abs. at 400 nm ^**^ (NaOH)
Soil A	Agricultural land	6.22	23.2	20.8	8.4	0.105	0.264
Soil B	Forest land	4.30	53.7	28.8	29.0	0.704	2.66

* EC: electrical conductivity; ** Abs. at 400 nm (TMAH and NaOH): absorbance values for 400 nm of alkaline soil extracts were obtained by mixing 1 g of soil, 4 mL of MUB, at pH 6.0, 0.25 mL of toluene, 1 mL of 0.5 M CaCl_2_, and either 4 mL of 0.1 M TMAH or 0.5 M NaOH, and the soil particles were removed.

The pH and electrical conductivity (EC) values for the soils were measured in slurries (1:2.5 air-dried soil/distilled water). The moisture content was determined from the loss in weight after drying at 105 °C for 48 h. These samples were also combusted at 600 °C for 2 h to determine the organic content by weight loss. Absorbance values for 400 nm of alkaline soil extracts were obtained by mixing 1 g of air-dried soil, 4 mL of MUB, at pH 6.0, 0.25 mL of toluene, 1 mL of 0.5 M CaCl_2_, and either 4 mL of 0.1 M TMAH or 0.5 M NaOH, and the soil particles were removed, and centrifuged at 4000 rpm (about 2000 g) for 5 min [22]. The soil particle-size fractions were separated by the sieving machine (MSV-1, AsOne, Tokyo, Japan).

### 2.3. Procedure for the Regulatory Soil Enzyme Assay

A regulatory assay for β-gal, β-glu and AcP was modified as reported by Tabatabai *et al.* and Eivazi *et al.* [6,8]. A soil sample (0.1 g) in a test tube was mixed with toluene (0.025 mL), and MUB (0.4 mL) at pH 6.0. After shaking the test tube, the substrate solutions (0.1 mL, 25 mM of PNPGal, PNPGlu and 115 mM of PNPP) was added to the test tube and incubated at 37 °C for 60 min. The enzyme reaction was terminated by adding 0.5 M CaCl_2_ (0.1 mL) and alkali solutions (0.4 mL, 0.1 M TMAH for β-Gal, β-Glu and 0.5 M NaOH for AcP) at pH 12. The soil particles and precipitate were removed, and centrifuged at 4000 rpm for 5 min. The absorbance values of 400 nm of PNP in the soil extracts were measured using a double beam spectrophotometer (U-2000A, Hitachi, Tokyo, Japan) equipped with a quartz UV-visible microcell. Control experiments for both the sample and substrate were carried out during each incubation step in order to measure the color developed using the substrates or dissolved organic matter. The control samples were composed of MUB (0.4 mL), CaCl_2_ (0.1 mL) and alkali solutions (0.4 mL) with soil (0.1 g). The control was composed of MUB (0.4 mL), CaCl_2_ (0.1 mL) and alkali solution (0.4 mL) with substrate solution (0.1 mL). The concentrations of PNP in the samples were estimated by subtracting the combined absorption values for the sample and the controls from the analytical samples. All results were expressed as mM PNP released·soil g^−1^·h^−1^.

### 2.4. Procedure for the Electrochemical Soil Enzyme Assay

Hydrodynamic amperometry was carried out using an ALS-1200 voltammetric analyzer (Bioanalytical Systems Inc. (BAS) West Lafayette, IN, USA) with a glassy carbon disk electrode (6.0 mm outer diameter, 3.0 mm inner diameter, BAS), Pt wire (0.5 mm diameter), and Ag wire (0.7 mm diameter) as the working, counter and reference electrodes, respectively. Prior to the measurement, the glassy carbon disk electrode was polished sequentially using 0.3 and 0.05 μm of alumina paste, and was then rinsed well with distilled water.

**Figure 1:**
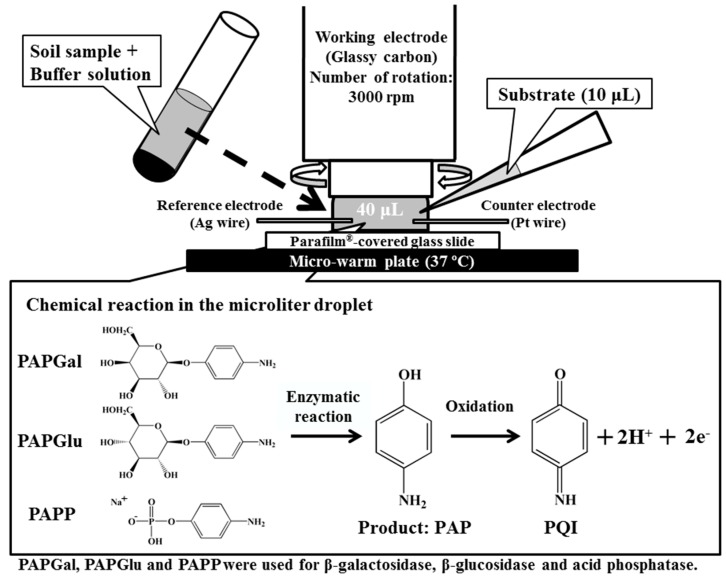
Schematic diagram of the enzyme assay with hydrodynamic voltammetry using a rotating disk electrode in a microdroplet. PAPGal: *p*-aminophenyl-β-D-galactopyranoside; PAPGlu: *p*-aminophenyl-β-D-glucopyranoside; PAPP: *p*-aminophenyl phosphate monosodium salt; PAP: *p*-aminophenol; and, PQI: *p*-quinone imine.

Schematic diagrams of the detection system using a RDE, to measure the enzymatic and electrochemical reactions are illustrated in Figure 1. Hydrodynamic chronoamperometry and linear sweep voltammetry were used for the evaluation of soil enzyme activity. In the chronoamperometry experiment, 40 μL of the soil extract was prepared using the same procedure as a regular assay (0.1 g soil + 0.4 mL MUB + 0.025 mL toluene), and was placed between the RDE and the Parafilm^®^-covered glass slide on a thermostatically controlled warm plate (KM-1, Kitazato Science Co. Ltd., Shizuoka, Japan) at 37 °C. The amperometric detection was performed by applying a potential of 0.3 V at a rotation rate of 3000 rpm. After an equilibration time of 20 s, the current was measured in order to establish a baseline. At 30 s, 10 μL of substrate solutions (PAPGal: 200 mM, PAPGlu: 400 mM, PAPP: 100 mM) were added to the droplet via micropipette, and the current recording was continued for an additional 60 s. The slope between 50 and 60 s was corrected by subtracting the background slope, taken between 10 and 20 s, to give the reaction rate. The linear sweep voltammetry using the RDE system was performed with soil, MUB, toluene and substrate mixture incubated at 37 °C. After 60 min, a 50 μL droplet of the reaction mixtures was sandwiched between the RDE and the Parafilm^®^-covered glass slide, and the hydrodynamic linear sweep voltammetry was done using a scan rate of 100 mV·s^−1^ with a rotation rate of 3000 rpm. The control for the sample consisted of 0.4 mL of MUB with 0.1 g of soil. The concentrations of PAP in the samples were estimated by subtracting the current values for the sample controls from the analytical samples.

## 3. Results and Discussion

### 3.1. The Effect of Colored Organic Matter and Soil Particles on the Hydrodynamic Voltammetric Detection for PAP

**Figure 2:**
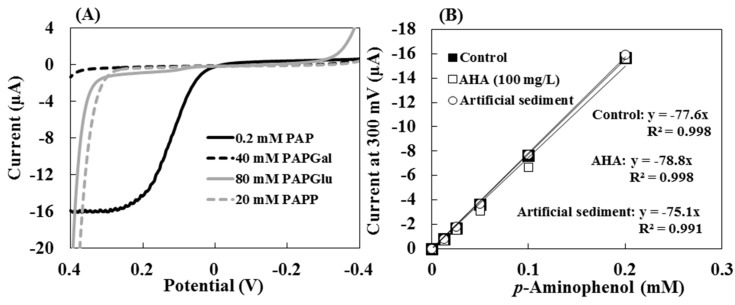
(**A**) The hydrodynamic linear sweep voltammograms of 0.2 mM PAP, 40 mM PAPGal, 80 mM PAPGlu and 20 mM PAPP in MUB, at pH 6.0 with 5% toluene; (**B**) The calibration curve of the mass-transfer limited current of PAP at 0.3 V without (control) or with 100 mg·L^−1^ AHA and artificial sediment. Symbols: ■ = Control, □ = with AHA, ○ = with artificial sediment. The measurements were accomplished in a droplet (50 μL) at a scan rate of 100 mV·s^−1^ with a rotation rate of 3000 rpm.

Figure 2A shows the hydrodynamic linear sweep voltammograms of 0.2 mM PAP and each substrate (40 mM PAPGal, 80 mM PAPGlu and 20 mM PAPP) in MUB, at pH 6.0 with 5% toluene. The measurements were performed at a scan rate of 100 mV·s^−1^ with a rotation rate of 3000 rpm and 20 s of quiet time. The linear sweep voltammetry was carried out at potentials that ranged from −0.4 to 0.4 V. The current response of the PAP increased in potentials at between 0 and 0.4 V, and the diffusion-limited current was observed at more than 0.25 V. However, the oxidation current of each substrate was obtained at more than 0.3 V. Thus, in this assay, a potential of 0.3 V was selected to detect the optimal amount of PAP released by an enzymatic reaction.

The calibration curve of the mass-transfer limited current of PAP in the presence of the 100 mg·L^−1^ AHA and artificial sediment (*Sphagnum* moss peat 4%, kaolin 20%, quartz sand 76%, particle size was ≤0.025 mm) are shown in Figure 2B. The calibration curves of the current for the oxidation of PAP at 0.3 V without (control) and with AHA and artificial sediment indicated similar slope values and a good linear relationship. These results indicated that the hydrodynamic voltammetric detection of PAP was not affected by the high concentration of colored dissolved organic matter, soil particles and toluene. Therefore, the application of hydrodynamic voltammetric detection to an enzyme assay can be used to evaluate samples that include high concentrations of organic matter and soil particles.

### 3.2. Optimization of the Substrate Concentration for Electrochemical Soil Enzyme Assay

**Figure 3:**
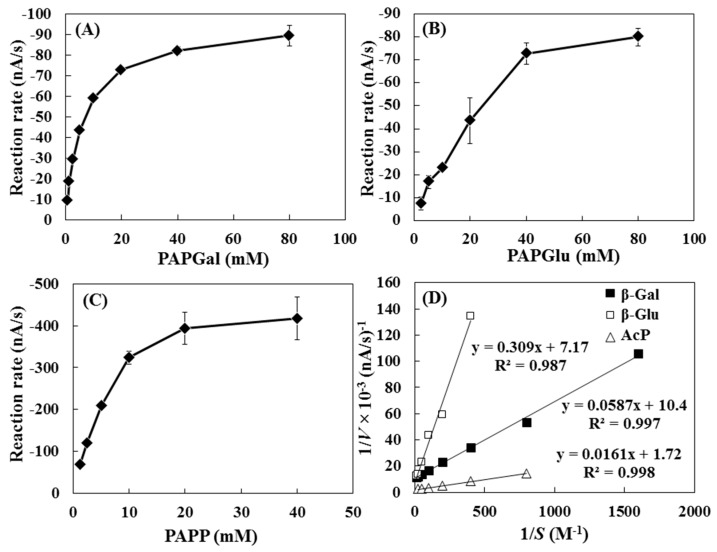
The hydrodynamic amperometric responses of (**A**) 0.0075 mg·mL^−1^ β-gal; (**B**) 10 mg·mL^−1^ β-glu and (**C**) 1.0 mg·mL^−1^ AcP with different concentrations of substrate at 0.3 V applied potential, with rotation set at 3000 rpm; (**D**) Lineweaver-Burk plots for β-gal, β-glu and AcP as obtained by hydrodynamic voltammetry; *V* is the absolute value of the amperometric response (nA·s^−1^). Error bars represent standard errors (*N* = 3).

The optimal concentration of substrate for the evaluation of enzyme activity in this method was investigated. The hydrodynamic amperometric responses of commercial grade enzymes ((A) 0.0075 mg·mL^−1^ of β-gal; (B) 10 mg·mL^−1^ of β-glu and (C) 1.0 mg·mL^−1^ of AcP) solutions with different concentrations of substrate are shown in Figure 3. In the regulatory assay, 0.0075 mg·mL^−1^ of β-gal, 10 mg·mL^−1^ of β-glu, and 1.0 mg·mL^−1^ of AcP solutions released 2.28 mM, 252 mM, and 23.8 mM, respectively, of PNP at 37 °C after 60 min. Ten μL of 200 mM PAPGal, 400 mM PAPGlu, and 100 mM PAPP were added to each 40 μL sample of enzyme solution, and the amperometric responses reached a plateau. The optimal concentrations of substrate for these enzymes with hydrodynamic chronoamperometry were chosen as follows: 40 mM PAPGal for β-gal; 80 mM PAPGlu for β-glu; and, 20 mM PAPP for AcP. Figure 3D shows the Lineweaver-Burk plots (1/*V vs.* 1/*S*) for β-gal, β-glu and AcP, as obtained by hydrodynamic voltammetry. The enzyme kinetic parameters, Michaelis-Menten constant (*K*_M_), and the maximum velocity (*V*_max_) were calculated from the intercept (*V*_max_) and the slope (*K*_M_/*V*_max_) of the Lineweaver-Burk plots. The *K*_M_ values were as follows: β-gal = 5.6 mM, β-glu = 43 mM, and AcP = 9.3 mM. The *V*_max_ values were as follows: β-gal = −100 nA·s^−1^, β-glu = −139 nA·s^−1^ and AcP = −588 nA·s^−1^. Tabatabai and Bremner have reported that the *K*_M_ value of potato AcP was 19.8 mM as the results from the regulatory assay [23]. From the *K*_M_ and *V*_max_ values, the proposed method is usable in evaluating AcP activity in soils.

### 3.3. Demonstration of Hydrodynamic Electrochemical Detection for Enzyme Activities in Laboratory-Spiked Soils Using the RDE System

**Figure 4:**
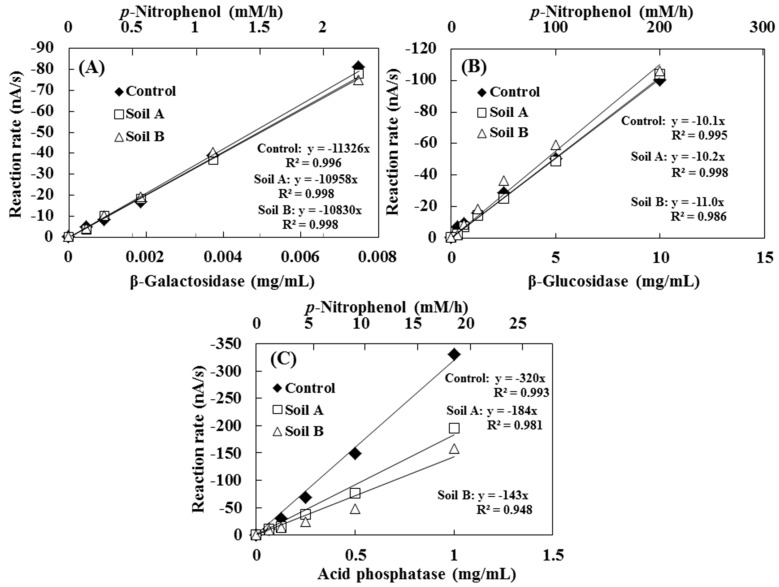
The calibration curve for (**A**) β-gal; (**B**) β-glu and (**C**) AcP solutions spiked in real soils. The original enzymes in the soil samples were inactivated in an autoclave at 121 °C for 20 min. The concentration of the *p*-nitrophenol released from the enzyme reaction was measured using standard assay methods.

The calibration curves for β-gal, β-glu and AcP activities with or without soil samples obtained by hydrodynamic chronoamperometry are shown in Figure 4. The enzyme activities in soil samples were determined by spiking different concentrations of enzymes in MUB, at pH 6.0 directly to soil samples A and B. Before spiking each of the enzyme solutions, the original enzyme activities in the samples were inactivated via autoclave at 121 °C for 20 min. The calibration curves of β-gal and β-glu with the samples showed similar slope values with the control experiments (Figure 4A,B). However, in the case of AcP, a decrease in the enzyme activity was observed in the enzyme solutions including the soil samples. Within a range of 0.1 to 1.0 g·mL^−1^, the AcP activity in the presence of soils A and B were 45% and 59% lower than activity without soil (Figure 4C).

Generally, soil enzymes bind to the surface of clay minerals and couple with humic colloid polyphenols. Clay minerals reportedly increase enzyme stability [24] and humic substances are known to decrease enzyme activity by inhibiting substrate access to the enzyme active site [25,26,27]. Allison has shown that commercial-grade AcP activity is increased by the presence of more than 4% allophane, whereas the activity of AcP is reduced by the presence of more than 4% humic acid, and the degree of interference is positively correlated with the humic acid concentration [28]. Based on the ignition loss and absorbance value at 400 nm of soil alkali extract (Table 1), the amount of colored dissolved organic matter of soil B was higher than that of soil A. Thus, decreases in the AcP in the presence of the soils obtained in the present study could have been due to the differences in the quantity of humic substances in the soil. The calibration curves for each of the enzymes with or without the soil samples obtained via hydrodynamic chronoamperometry indicated a good linear correlation (control: *r* = 0.993 − 0.996; soil A: *r* = 0.981 − 0.998; soil B: *r* = 0.948 − 0.998). In addition, the percentage contributions (%soil dry wt.) of each particle-size fraction in soil A and B were as follows; Soil A: > 2–0.212 mm = 62.7%, 0.212–0.02 mm = 35.7%, <0.02 mm = 1.6%, Soil B: >2–0.212 mm = 77.4%, 0.212–0.02 mm = 21.6%, <0.02 mm = 1.0%. Namely, the main particle-size fraction of the soils showed 10-fold larger than the particle size of artificial sediment. It is clear that the presence of soil particle smaller than 2 mm hardly interfered with the electrochemical detection of PAP. The results obtained in present study showed that the proposed method was successful in evaluating the current response obtained from the oxidation of the PAP that was produced by the enzyme reaction in the soil extract solution.

### 3.4. The Demonstration for Determination of β-Galactosidase, β-Glucosidase and Acid Phosphatase Activity in Real Soils

**Figure 5:**
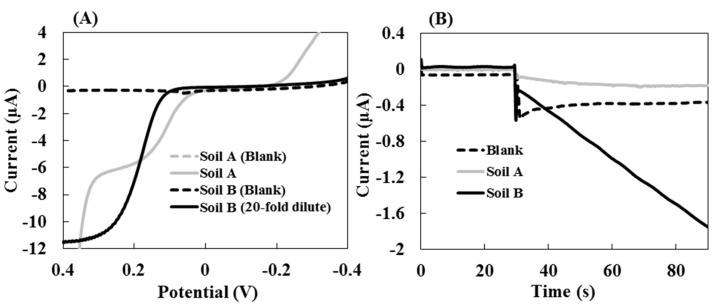
The AcP activity of real soil samples obtained from hydrodynamic (**A**) linear sweep voltammetry and (**B**) chronoamperometry. The linear sweep voltammogram of the soil B mixture was diluted 20-fold with MUB, at pH 6.0, after incubation for 60 min at 37 °C. The chronoamperogram measurements were performed using the RDE system at 0.3 V applied potential with a rotation rate of 3000 rpm.

The activities of each of the enzyme in real soils were assessed using standard and electrochemical detection methods. Figure 5 shows the AcP activity in soils A and B obtained from linear sweep voltammetry and hydrodynamic chronoamperometry. In the linear sweep voltammetry experiments, after incubating the soil mixture with substrate for 60 min at 37 °C, the mass-transfer limited currents of PAP released from the AcP in soil B was higher than that in soil A (Figure 5A). In this assay, an incubated mixture of soil B for AcP was diluted 20-fold with MUB, at pH 6.0, because the current value of PAP at 0.3 V in soil B exceeded the range of the PAP calibration curves, as shown in Figure 2. In addition, the background signal the current values of the soil mixtures without substrate were unchanged from −0.4 V to 0.4 V. The chronoamperometric signals from the oxidation of the PAP released from the AcP in the soil samples were detected (Figure 5B: the reaction rate, soil A = −1.27 ± 0.36 nA·s^−1^, soil B = −29.8 ± 7.5 nA·s^−1^, the values are reported as the means (*N* = 3)).

**Figure 6:**
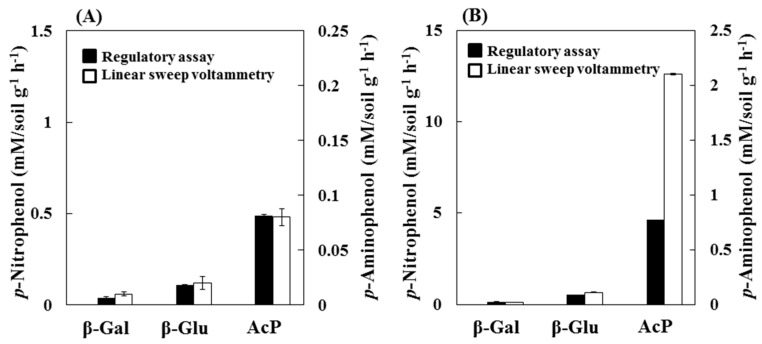
The β-gal, β-glu and AcP activities of (**A**) soil A and (**B**) soil B obtained from regulatory assay and linear sweep voltammetry. Regulatory assay: mM PNP·soil g^−1^·h^−1^ at 37 °C, Linear sweep voltammetry: mM PAP·soil g^−1^·h^−1^ at 37 °C. Error bars represent standard errors (*N* = 3).

The results obtained from chronoamperometry showed that the ratio of the AcP activity of soils B and A were almost the same as the results from the linear sweep voltammetry (linear sweep voltammetry = 26 and chronoamperometry = 23). Therefore, chronoamperometry is a useful tool that enables the easy and rapid evaluation of the enzyme activity in soil, the measurement was completed in 90 s; however, β-gal and β-glu activities were not detected in either of the soil samples. From the *V*_max_ values of commercial enzymes obtained by hydrodynamic chronoamperometry, the enzymatic reaction rates of β-gal and β-glu were lower than that of AcP. Therefore, it seems that the evaluation of β-gal and β-glu activities in the soils requires a long incubation time. The β-gal and β-glu activities of the soil samples were evaluated based on the PAP produced by an enzymatic reaction after 60 min using linear sweep voltammetry. The soil enzyme activities obtained by spectrophotometric and linear sweep voltammetry are shown in Figure 6.

With the use of standard assay, an incubated mixture of both soil for AcP and soil B for β-glu were diluted 10- and 40-fold using distilled water (AcP of soil A and β-Glu of soil B = 10-fold, AcP of soil B = 40-fold), because the absorbance value of the PNP in the soil extract solutions exceeded the range of the calibration curves. Spectrophotometric detection was more affected by colored dissolved organic matter than by electrochemical detection. The results of the electrochemical detection were expressed as mM PAP released·soil g^−1^·h^−1^, which was calculated from the calibration curve of the PAP. Each of the enzymes in both soil samples was evaluated by standard assay and linear sweep voltammetry, and each indicated the same order (AcP > β-glu > β-gal). In addition, all enzyme activities were higher in soil B than in soil A. In a previous report, some enzyme activities were positively correlated with the soil organic carbon content. The β-glu and AcP activities were higher in forest soil than in agricultural soil [29]. Based on the results from soil A, the enzyme activities determined by this method are comparable to those obtained from a regulatory assay. With regards to soil B, the intensity of the AcP activity obtained from a electrochemical detection was higher than that obtained via standard assay. The color of the PNP released by the AcP activity in soil B seemed to be significantly influenced by the presence of a high concentration of colored dissolved organic matter in the NaOH extract (Table 1).

This study clearly showed that the hydrodynamic electrochemical method requires no filtration procedure to remove soil particles. For the evaluation of soils that contained a high concentration of colored organic matter and enzymatic activity, this method is more suitable than the standard assay method. Therefore, this new soil enzyme assay has the potential to be utilized for the rapid monitoring of soil health and quality.

## 4. Conclusions

A new soil enzyme assay based on hydrodynamic electrochemical detection in a microliter droplet using the RDE system was developed. The results from the laboratory-spiked samples, showed the chronoamperometry calibration curves for β-gal, β-glu and AcP, showed good linear correlation with the results of the control experiments, even in the presence of high concentrations of colored dissolved organic matter and soil particles. Since the developed enzyme assay was not affected by the existing colored dissolved organic matter and soil particles, unlike a regulatory assay no filtration procedure was required to remove soil particles. Furthermore, the detection time for this method was only 90 s; thus the AcP activity of real soils can be detected by chronoamperometry. Also, we evaluated soil enzyme activities using linear sweep voltammetry, and were able to assess the amount of PAP released by an enzymatic reaction after 60 min. The enzyme activities in real soils based on spectrophotometric and linear sweep voltammetry showed good agreement. The findings from this study suggest that the proposed hydrodynamic electrochemical method can be used as a rapid, simple procedure that is user-friendly.
